# Multifunctional TRPV1 Ion Channels in Physiology and Pathology with Focus on the Brain, Vasculature, and Some Visceral Systems

**DOI:** 10.1155/2019/5806321

**Published:** 2019-05-27

**Authors:** Maksim V. Storozhuk, Olesia F. Moroz, Alexander V. Zholos

**Affiliations:** ^1^A.A. Bogomoletz Institute of Physiology, National Academy of Sciences of Ukraine, 4 Bogomoletz Street, Kiev 01024, Ukraine; ^2^Taras Shevchenko National University of Kyiv, Educational and Scientific Centre “Institute of Biology and Medicine”, 2 Academician Glushkov Avenue, Kiev 03022, Ukraine

## Abstract

TRPV1 has been originally cloned as the heat and capsaicin receptor implicated in acute pain signalling, while further research has shifted the focus to its importance in chronic pain caused by inflammation and associated with this TRPV1 sensitization. However, accumulating evidence suggests that, apart from pain signalling, TRPV1 subserves many other unrelated to nociception functions in the nervous system. In the brain, TRPV1 can modulate synaptic transmission via both pre- and postsynaptic mechanisms and there is a functional crosstalk between GABA receptors and TRPV1. Other fundamental processes include TRPV1 role in plasticity, microglia-to-neuron communication, and brain development. Moreover, TRPV1 is widely expressed in the peripheral tissues, including the vasculature, gastrointestinal tract, urinary bladder, epithelial cells, and the cells of the immune system. TRPV1 can be activated by a large array of physical (heat, mechanical stimuli) and chemical factors (e.g., protons, capsaicin, resiniferatoxin, and endogenous ligands, such as endovanilloids). This causes two general cell effects, membrane depolarization and calcium influx, thus triggering depending on the cell-type diverse functional responses ranging from neuronal excitation to secretion and smooth muscle contraction. Here, we review recent research on the diverse TRPV1 functions with focus on the brain, vasculature, and some visceral systems as the basis of our better understanding of TRPV1 role in different human disorders.

## 1. Introduction

“Transient receptor potential” (TRP) channels are a superfamily of about 28 nonselective cation channels divided into 7 subfamilies including TRP vanilloid (TRPV) [1]. Channels of this superfamily display greater diversity in the activation mechanisms, voltage dependence, selectivity, and pharmacological properties than any other class of ion channels [1].

TRPV1 receptor (transient receptor potential vanilloid subfamily, member 1), initially described as a specific target of capsaicin and resiniferatoxin [2], was cloned in 1997 from the rat dorsal root ganglia (DRGs) [3]. It immediately caught substantial theoretical and practical interest since it was appropriately highlighted as “a heat-activated ion channel in the pain pathway” in this original paper. Besides capsaicin, TRPV1 can be activated by many physical and chemical stimuli including noxious heat (>43°C), low extracellular pH, and putative endovanilloids [4].

Considering that TRPV1 channel is predominantly expressed in neurons related to nociception, most of the earlier studies on TRPV1 were related to its role in nociception, accordingly pharmacological intervention targeting TRPV1 was primarily aimed at treating pain. Nevertheless, already in 2007, it became apparent that TRPV1 is also expressed in neurons not related to nociception as well as in many different nonneuronal tissues, implying that “TRPV1 is more than a pain sensor”[4]. In this regard, rather widespread presence of TRPV1 in brain neurons (reviewed in [5, 6], but see, for instance, [7] for controversial results) and its functional role there raise many challenging questions.

At present, the structure of TRPV1 protein has been determined by electron cryomicroscopy [8]; moreover combining electron cryomicroscopy with lipid nanodisc technology allowed ascertaining the structure of TRPV1 ion channel in a native bilayer environment [9]. Currently, TRPV1 is implicated in multiple physiological and pathophysiological processes including pain [10]; thermosensation [11]; energy homeostasis [12]; modulation of autophagy and proteasome activity [13]; reciprocal crosstalk between the sensory nervous and immune systems [14]; regulation of diet-induced obesity; insulin and leptin resistance [15]; cancer [16, 17]; the development severe bronchial asthma [18]; and even in itch and inflammation [19].

Here, we will review recent research on the diverse TRPV1 functions with focus on the brain, vasculature, and some visceral systems as the basis of our better understanding of its role in different human disorders. The reason for this focus is* relative* lack of interest in these issues in the literature. In the first section, we only briefly outline some of the most recent findings regarding TRPV1 and nociception and then focus on the emerging concepts regarding other roles of this receptor in the brain.

## 2. Some of the Most Recent Findings Regarding the Role of TRPV1 in Nociception

It has been shown that that acute noxious heat sensing in mice depends on a* triad* of TRP ion channels (TRPM3, TRPV1, and TRPA1) [20]. Indeed,* Trpv1*^−/−^*Trpm3*^−/−^*Trpa1*^−/−^-triple knockout mice lack the acute withdrawal response to noxious heat, while showing normal nociceptive responses to cold or mechanical stimuli. Nevertheless, robust somatosensory heat responsiveness can still be observed at the cellular and behavioral levels if at least one of these receptors is functional [20].

Another recent work suggests that TRPA1 nociceptive responses in human skin strongly depend on intact capsaicin-sensitive, TRPV1^+^ fibers [21]. In their work, Nielsen and colleagues investigated whether functional responses from the subpopulation of TRPA1^+^ nociceptors could be evoked after defunctionalization of TRPV1^+^ nociceptors by cutaneous application of high-concentration capsaicin. It has been found that ablation of cutaneous capsaicin-sensitive afferents caused consistent and equal inhibition of both TRPV1- and TRPA1-provoked responses assessed psychophysically and by imaging of vasomotor responses [21].

Hanack and colleagues [22] have shown that GABA_B1_ receptor subunit inhibits TRPV1 sensitization. This action is mediated by* noncanonical*  GABA_B_ pathway, and most notably it is independent of G protein signaling. Instead, it relies on a close juxtaposition of GABA_B1_ and TRPV1. Importantly, activation of GABA_B1_ selectively affects the sensitized state of TRPV1 channels implicated in pathological pain, but leaves acute TRPV1 pain signaling intact. Moreover, the native agonist of GABA_A_ and GABA_B_ receptors is* endogenously* present at* peripheral nerve endings *to produce a basal GABA_B_ receptor activity that regulates TRPV1 sensitivity [22]. Thus, peripheral alteration of GABA_B_ receptor tone is a promising approach for developing analgesics [22].

Interestingly, several other recent studies also support important role of* endogenous *GABA and* peripheral* GABA receptors in processing nociceptive signaling [23, 24]. Moreover, there is an interaction between TRPV1 and GABA_A_ receptor via GABA_A_ receptor associated protein [25] and TRPV1 plays important role in GABAergic neurons [26]. Together with other data indicating functional crosstalk between GABA and TRPV1 (see [27, 28] for review), the results outlined above suggest that GABA agonists (as well as GABA itself) may be used to affect TRPV1 functioning.

Regarding approaches of targeting TRPV1, it is worth mentioning the recent finding by Korolkova and coauthors showing that low-molecular-weight compounds isolated from marine sponge* Monanchora pulchra *have inhibitory effect on several TRP channels including TRPV1 [29].

## 3. TRPV1 in the Brain

### 3.1. Physiological Role of TRPV1 in the Brain

As already mentioned, functional role of TRPV1 in the brain is a challenging question. In particular, since large variations in temperature and pH are unlikely to occur in the brain, it was not clear for a while: what activates TRPV1 in this structure under physiological conditions? It seems that the answer is that these are endogenous vanilloids/cannabinoids (see [30, 31] for review). Changes of the extracellular levels of endogenous vanilloids/cannabinoids, in particular, induced by neuronal activity may activate neuronal TRPV1 and thus modulate synaptic strength. Among putative endovanilloids, three different classes of endogenous lipids have been identified so far: (i) unsaturated N-acyldopamines, (ii) lipoxygenase products of arachidonic acid, and (iii) the endocannabinoid anandamide with some of its congeners [30]. It is also worth mentioning that TRPV1 (and some of the other members of the TRP superfamily of ion channels) is suggested to be considered as “ionotropic cannabinoid receptor” by some authors [32–34]. Therefore, in addition to anandamide, other endocannabinoids may also act as endovanilloids.

Many studies on the role of TRPV1 channels in the brain have focused on their role in the regulation of synaptic transmission. By now, it is well documented that activation of TRPV1 can modulate synaptic transmission via both pre- and postsynaptic mechanisms. For instance, it has been concluded that TRPV1 is located presynaptically on afferents to the* locus coeruleus* and that activation of this receptor potentiates the release of glutamate and adrenaline/noradrenaline in this brain region [35]. Similarly, in striatum, the effect on glutamatergic transmission was shown to be presynaptic [36].

On the other hand, TRPV1 suppressed the excitatory transmission in rat and mouse dentate gyrus via postsynaptic mechanism, namely, Ca^2+^-calcineurin and clathrin-dependent internalization of AMPA receptors [37]. In the nucleus accumbens, TRPV1-dependent depression of the excitatory transmission is also mediated by a postsynaptic mechanism, such as endocytosis of AMPA receptors [38].

In addition to modulation of glutamatergic transmission, TRPV1 can be also involved in the modulation of GABAergic transmission [39]. For instance, TRPV1 activation by capsaicin or by the endocannabinoid anandamide depresses somatic, but not dendritic inhibitory GABAergic transmission in both rat and mouse dentate gyrus [40]. Specificity of the effects was further confirmed by experiments using TRPV1 knockout mice. The mechanism of the TRPV1-mediated depression of GABAergic transmission is postsynaptic (likely due to clathrin-dependent internalization of GABA receptors) [40].

Very recent studies further supported the important role of TRPV1 in the regulation of synaptic transmission in different brain structures. For instance, the involvement of TRPV1 in spike-timing-dependent LTP (tLTP) in neocortical pyramidal cells has been demonstrated [41], suggesting that this receptor may be of importance for acquiring new associative memories [41]. Block of TRPV1 dramatically reduces glutamatergic input to oriens-lacunosum-moleculare (OLM) interneurons in the hippocampus [42]. Besides, TRPV1 plays* synaptogenic* role in a specific interneuron population in the hippocampus, as revealed in experiments using TRPV1 knockout mice [42]. Important role of TRPV1 in the regulation of synaptic transmission in the hippocampus and development/maturation of this structure is also strongly supported by direct pharmacological evidence for the* functional *expression of TRPV1 channels in hippocampal Cajal-Retzius cells [43]. As Cajal-Retzius cells powerfully excite GABAergic interneurons of the molecular layers, TRPV1 could play important roles in the regulation of layer-specific processing [43]. Additionally, considering that Cajal-Retzius cells are a major source of the extracellular matrix protein reelin, which is essential for development (see [44] for a review), these results further support potential importance of TRPV1 in brain development.

Although numerous studies showed the involvement of TRPV1 in the regulation of synaptic transmission in the brain, recent work by Marrone and coauthors [45] challenged the idea that this regulation is the result of activation* of neuronal* TRPV1. Indeed, this group provided evidence indicating that modulation of synaptic transmission is the result of activation of TRPV1 in* microglia*. According to the observations of this group, TRPV1 is highly expressed in microglial cells* rather than neurons* of the anterior cingulate cortex* and* other brain areas. Moreover, stimulation of microglial TRPV1 controls cortical microglia activation* per se* and* indirectly* enhances glutamatergic transmission in neurons [45]. Another interesting finding of this group is that neuropathic pain results in a dramatic enhancement of functional expression of TRPV1 in brain neurons (although in control animals, the expression of TRPV1 is nearly nondetectable in neurons of most of the brain areas). Altogether, these findings allowed the authors to conclude that “brain TRPV1 is a potential detector of harmful stimuli and a key player of microglia-to-neuron communication” [45].

In any case, what is above mentioned indicates that (even) within the brain, TRPV1 is of great functional importance for several fundamental processes, including modulation of synaptic transmission and its plasticity, microglia-to-neuron communication, and development. Thus, it is not surprising that TRPV1 is currently implicated not only in pain transduction, but also in several neurological and psychiatric disorders such as epilepsy, anxiety, and depression as well as drug-addiction disorders [6].

### 3.2. TRPV1 and Brain Disorders

#### 3.2.1. Epilepsy

It has been reported that expression of TRPV1 is increased in the dente gyrus of mice exhibiting pilocarpine-induced limbic status epilepticus [46] and in the cortex and hippocampus from patients with mesial temporal lobe epilepsy [47]. These observations suggest that TRPV1 inhibition should suppress limbic seizures. In line with this, inhibition of TRPV1, using its antagonist AMG-9810 [(E)-3-(4-t-butylphenyl)-N-(2,3-dihydrobenzo[b][1,4] dioxin-6-yl)acrylamide], prevented the development of clonic and tonic-clonic seizures following amygdala kindling [48]. *α*-Spinasterol, another TRPV1 antagonist, elevated the seizure threshold in three acute seizure tests in mice [49]. Additionally, inhibition of TRPV1 by capsazepine suppressed seizure susceptibility in the genetically epilepsy-prone rat [50].

On the other hand,* agonist* of TRPV1 capsaicin suppressed kainic acid-induced limbic status epilepticus [51]. The controversy with the results mentioned above, however, may be explained by the desensitizing action of capsaicin on TRPV1. Nevertheless, such an explanation is not valid for antiseizure effects of another agonist of TRPV1—piperine [52], since these were blocked by capsazepine. Results of the very interesting recent work of Suemaru and coauthors [53], probably, also should be interpreted as supporting anticonvulsant effects of TRPV1* agonists. *They have reported that (i) anticonvulsant effects of acetaminophen are similar to that of one of its active metabolites AM404; (ii) anticonvulsant effects of acetaminophen are blocked by TRPV1 antagonists capsazepine and AMG9810, but still observed in the presence of CB1 receptor antagonist AM251. Therefore, considering that AM404 is an inhibitor of the uptake of the endocannabinoid/endovanilloid anandamide, it seems likely that activation of TRPV1 is responsible for the anticonvulsant effects.

A related point to consider regarding the controversies is as follows. Since activation of TRPV1 can substantially (more than two times) change neuronal firing [54] and the effect has rather slow onset latency (~5 minutes) [54], it is worth mentioning that* prolonged *alteration of activity in neuronal networks initiates a number of homeostatic mechanisms including compensatory changes of synaptic strength and plasticity [55–59]. Thus, it cannot be excluded that an effect of TRPV1 activation is mediated/counterbalanced by the homeostatic mechanisms* per se*.

In any case, there are still some controversies regarding beneficial effects of TRPV1 activation/inhibition as potential antiepileptic treatments.

#### 3.2.2. Depression

Pharmacological studies as well as experiments on TRPV1 knockout mice suggest an important role of this receptor in depressive disorder (persistent and unreactive low mood or loss of interest and pleasure) (see [60] for a review). In particular, experiments on TRPV1 knockout mice suggest that block of this receptor causes antidepressant effect [61], while its pharmacological activation increases depressive behavior [62].

#### 3.2.3. Schizophrenia

“Schizophrenia is a chronic psychiatric disorder which causes lifelong disability, resulting in major individual and societal cost” [63]. There is growing evidence suggesting potential role of TRPV1 in schizophrenia (see [28, 60, 63] for review). Here, we will mention just some notable findings: the presence of TRPV1 in dopaminergic neurons and its functional role in the regulation of dopamine release together with antipsychotic efficacy of dopamine D_2_ receptor antagonists [63]; results of psychopharmacological studies indicating that TRPV1 modulates behavioral changes in schizophrenia models [64, 65].

#### 3.2.4. Alzheimer's Disease

It has been recently reported that activation of TRPV1 in rodents protects neurons from cytotoxic effects of Amyloid-*β* peptide (A*β*) and reverses hippocampal damage and memory impairment [66]. Indeed, both effects of capsaicin were blocked by capsazepine and absent in* Trpv1* knockout mice [66], thus implying that targeting TRPV1 may be useful for the treatment of Alzheimer's disease.

There is also evidence that alteration of TRPV1 functioning affects* drug addiction* [67–69].

Thus, considering the brain alone, TRPV1 is clearly a multifunctional channel implicated in multiple physiological and pathophysiological processes. In developing any new strategy for treating human disorders, one will necessary need to address the whole scope of the potential benefits as well as drawbacks of TRPV1 pharmacological targeting (see [70] for a review).

## 4. TRPV1 in Vascular and Visceral Systems

TRPV1 is best known to be thermo-, mechano- and capsaicin-sensitive cation channel mediating the sensation of burning heat and pain. Out of the brain, TRPV1 is mostly expressed in sensory fibers that originate in the dorsal root, trigeminal or vagal ganglia [71]. TRPV1 is also found in perivascular sensory neurons, in the plasma membrane of keratinocytes, in the cells of the immune system, and in smooth muscle cells and urothelium [72]. In the urinary bladder, TRPV1 appeared to mediate stretch-evoked ATP release indicating its role as mechanosensor [73]. In blood vessels, the increase of intraluminal pressure causes ligand-dependent activation of TRPV1 [74]. In peripheral tissues, where tissue temperature is not subject to any significant variations, TRPV1 is supposed to be gated by protons that accumulate under conditions of inflammation, oxidative stress, and ischemia [75], several arachidonic derivates such as 20-hydroxyeicosateraenoic acid (20-HETE) [76], 5- and 15-(S)-hydroxyeicosatetraenoic acids, 12- and 15-(S)-hydroperoxyeicosatetraenoic acids (HPETE), 2-arachidonylglycerol [71], N-arachidonoyl dopamine (NADA) [77], and also by anandamide [78, 79]. Activity of TRPV1 is modulated by protein kinases A and C and phosphorylation of the channel by Ca^2+^-calmodulin-dependent kinase II is crucial for its ligand binding [78]. Visceral systems that are reviewed here for the role of TRPV1 are selected taking into account some common features, like functional similarity (smooth muscle layer of different organs), common origin (endothelium and other epidermal tissues like keratinocytes), or integrative role in the organism (like immune cells or nervous system).

### 4.1. TRPV1 in Vascular Disorders

TRPV1 channels are expressed on endothelial cells, smooth muscle cells, and perivascular nerves [80]. They are involved in the regulation of vascular tone, blood pressure and play significant role in cardiovascular pathology [81, 82]. Participation of TRPV1 expressed in perivascular nerves in the regulation of circulation is likely based on the same mechanism, through which these channels are acting in the brain, which makes it possible to consider them as common calcium-dependent regulatory pathways, typical for both the central nervous system and viscera.

In the resistance arteries, TRPV1 mediates myogenic vasoconstriction in the Bayliss reflex. Increased intraluminal pressure triggers 20-hydroxyeicosatetraenoic acid formation in vascular smooth muscle cells (VSMC) that stimulates TRPV1 on sensory nerve endings leading to nerve depolarization and release of substance P. Interaction of this neuropeptide with NK1 tachykinin receptor on VSMC finally triggers the contractile response [74]. TRPV1 channels, when gated by capsaicin and anandamide, mediate hypotensive effect in the cardiovascular system. When these receptors are activated in sensory neurons, calcitonin gene-related peptide release is increased that leads to vasodilation and a drop in blood pressure. TRPV1 agonists stimulate phosphorylation of protein kinase A and endothelial nitric oxide synthase (NOS) [83]. Stimulation of endothelial TRPV1 causes relaxation of vascular smooth muscle cells via NO and potassium dependent pathways together with the involvement of endocannabinoids and their specific receptors [80, 84]. Similar mechanisms including NO synthase and Ca^2+^-dependent PI_3_K/Akt pathway are involved in endothelium protective action of TRPV1. Lipopolysaccharide-induced cytokine and chemokine production as well as adhesion molecule expression, activation of NF-*κ*B, and monocyte adhesion were reduced under capsaicin treatment [85]. That is why agonists of TRPV1 can be used both to control and to prevent hypertension and endothelium inflammation. Sun and colleagues in their review article mention that the long-term capsaicin treatment attenuates excess salt consumption-induced vascular dysfunction due to the inhibition of vascular oxidative stress [86]. Consumption of 0.01% capsaicin for 6 months significantly increased levels of p-eNOS in mesenteric arteries from wild-type mice in comparison with nontreated mice, but not in* TRPV1*^−/−^ mice suggesting that endothelial TRPV1 activation increases Ca^2+^-dependent phosphorylation of eNOS at Ser^1177^ and consequential vasodilatation [84]. Taking into account that TRPV1 channels are involved in the signaling pathways mediating the endothelium-derived or myogenic mechanisms of regulation of vascular tone and consequently blood pressure, these channels could be considered to affect this way contractility phenotype of myocardial cells. The latter is known to be dependent upon (i) the filling pressure and volume (preload) that could overstretch myocardial cells triggering Frank-Starling mechanism; (ii) the vascular resistance that should be overcome by systolic contraction (afterload) leading to cardiac hypertrophy. This way, TRPV1-mediated changes of vascular diameter are involved in myocardial functioning [87].

TRPV1 have also been shown to be involved in the pathogenesis of pulmonary hypertension—a disorder that could be developed under chronic hypoxia and leads to right heart failure and death. Experiments on rat pulmonary artery smooth muscle cells (PASMC) indicate that hypoxia promotes TRPV1 activation that could be a result of conformation change within the channel protein or due to the alteration in the concentration of endogenous lipid-derived molecules or because of an increase in the channel migration to the PASMC plasma membrane [88]. Experiments with caffeoylquinic acid (CQA) derivatives, isolated from* L. fischeri*, have demonstrated anti-inflammatory effect under hypoxic conditions acting on TRPV1-mediated pathways [89]. The study of idiopathic pulmonary arterial hypertension (IPAH) pathogenesis revealed that vasoconstriction due to PASMC contraction and pulmonary vascular remodeling as the result of increased PASMC proliferation, growth, and migration are developed because of upregulation of TRPV1 channels. Thus, special antagonists of these channels as well as the suppressors of gene expression of TRPV1 may be developed as the potential treatment for patients with IPAH [90–92]. Dubes and coauthors showed that TRPV1 channels are one of the mediators of intracellular Ca^2+^ increase in PASMC under silicium oxide nanoparticles loading [93].

TRPV1 displays a preventive role in atherosclerosis development. These channels, when activated, cause an increase in ATP-binding cassette transporter A1 (ABCA1) expression in VSMC, which in turn cause higher cellular cholesterol cleavage. The intrinsic mechanism of this effect is calcium and protein kinase A-dependent. However, experiments using TRPV1 knockout mice have not demonstrated this beneficiary effect.

In case of high-fat diet, TRPV1 could be a therapeutic target for attenuation of atherosclerosis development [94]. Activation of TRPV1 by capsaicin impedes foam cells formation from VSMCs loaded with oxidized low-density lipoprotein (oxLDL). Mechanism underlying this effect includes maintaining of autophagy. Capsaicin promotes LC3II/LC3I ratio and beclin-1 level that are decreased under oxLDL as well as the expression of LAMP-1 and the number of lysosomes. It is suggested that activation of TRPV1 enhances autophagy through activating AMPK signaling pathway probably via increased cytosolic Ca^2+^ [95, 96].

### 4.2. TRPV1 in Visceral Disorders

The role of TRPV1 in the regulation of airway tone and reflexes is based on capsaicin-induced depolarization of vagal sensory fibers, which triggers reflexes causing increased smooth muscles contractility and interleukins released from respiratory endothelium [97]. Alterations in the expression of the channels are associated with the onset of some airway disorders, such as asthma and cough [98] (McGarvey et al., 2014). Their functioning has also been reported to be changed under oxidative stress, hypoxia, inflammation, or mechanical stretch in the airways [99]. In clinical trial antagonist of channels, XEN-D0501 has demonstrated beneficial effect for refractory, but not spontaneous cough treatment [100]. Recent studies also revealed the reduction of TRPV1 mediated type 2 T helper cytokines, epithelial cell-derived cytokines decrease together with the reduction of goblet cell hyperplasia, normalization of *α*-smooth muscle actin, and collagen deposition in the presence of capsazepine in murine chronic asthma model [101].

In gastrointestinal tract, TRPV1 channels that are expressed on vagal and spinal afferent neurons in the esophagus, stomach, and intestine are intensively investigated as putative targets for gastroesophageal reflux disease, gastric pain hypersensitivity, inflammatory bowel disease, and some other human disorders [102]. Modulation of TRPV1 function by altered expression, enhanced activation, or decreased activation threshold have been described in visceral hypersensitivity [103]. Despite the fact that TRPV1 antagonists have significant side effects (hyperthermia, afferent nerves desensitization), capsaicin ingested chronically (5 weeks) promoted significant reduction in visceral pain in volunteers with functional dyspepsia [104]. On the other hand, in patients with irritable bowel syndrome (IBD), rectal hypersensitivity was higher in response to capsaicin comparatively to healthy volunteers, but the expression of TRPV1 was the same, which indicates that increased channels sensitization can play a role in IBD-provoked visceral pain [105]. Wouters and coauthors revealed that such a sensitization could be mediated by histamine H_1_ receptors; thus, their inhibitors are investigated further as a new therapeutic strategy [106]. In chronic stress,* Trpv1 *promoter and expression of the TRPV1 receptor are increased indicating that upregulation of TRPV1 could be a cause of hypersensitivity in IBD [79].

Besides, sensory function of TRPV1 has been implicated in the stimulation of mucus secretion in the gut by enhancing mucosal blood flow due to vasodilatory effect [107]. TRPV1 also provides a control of motor function of the GI tract. Transient and long-lasting contractions were recorded in experiments using guinea-pig esophagus, ileum and murine distal colon, and rectum. They developed because of transmitters release from sensory nerves, which stimulate myenteric cholinergic neurons that result in contraction of smooth muscle. But the long-lasting capsaicin response in the lower GI tract appeared to depend also on neurotransmitters released from extrinsic sensory nerve endings [108]. Nevertheless, TRPV1 agonists significantly inhibit tone and movements of human intestinal preparations, which could be mediated by nitric oxide and/or vasoactive intestinal polypeptide [109].

Experiments on high-fat diet mouse indicate the impairment of TRPV1 response to mechanic stretch as the cause of overeating and obesity [110]. Thus, TRPV1 is in focus of new treatment approaches development [107] and recent data suggest both natural [111, 112] and synthetic [113] substances that affect TRPV1 as a potent treatment of various gastrointestinal disorders.

In the urinary tract, TRPV1 is present not only in sensory nerve fibers, but also on the urothelium and smooth muscle cells of the bladder [114]. Here, TRPV1 mediates, at least in part, mechanosensation of the bladder during its filling, but little is known if these channels could interact with purinergic P2X receptors modulating ATP release from the urothelium and ATP-sensitivity of the afferent fibers [115]. TRPV1 expression appears to be altered in diabetic bladder dysfunction [116]. Capsaicin and resiniferatoxin, which cause desensitization of TRPV1, were used to treat neurogenic detrusor overactivity, but together with channel antagonists like GRC-6211 that reduces bladder contraction frequency, these demonstrated significant side effects [117].

### 4.3. TRPV1 in Metabolic Disorders

TRPV1-positive neurons are found in adipose and pancreatic tissues. Thus, they are considered to play a certain role in metabolism control. In rodent models of type II diabetes, capsaicin application promoted chronic release of calcitonin gene-related peptide that led to impaired insulin secretion, while capsaicin-induced desensitization has been shown to improve insulin secretion in response to food intake [118]. TRPV1-mediated inflammation of pancreatic islet cells together with its facilitation of glucose-like peptide-1 secretion in the gut illustrates the new perspectives for use of TRPV1 modulators in diabetes therapy [119]. Activation of TRPV1 reduced plasma level of triglyceride and visceral fat mass by promoting PPAR*α*, UCP2, and adiponectin genes expression followed by activation of thermogenesis and energy expenditures [120]. That is why TRPV1 agonism is proposed to be used as a new approach to attenuate diabetes-induces obesity [121] and such effect of chronic capsaicin intake (f.i. 10mg/kg for 3-4 weeks) is supported by clinical trials [122].

**Figure 1:**
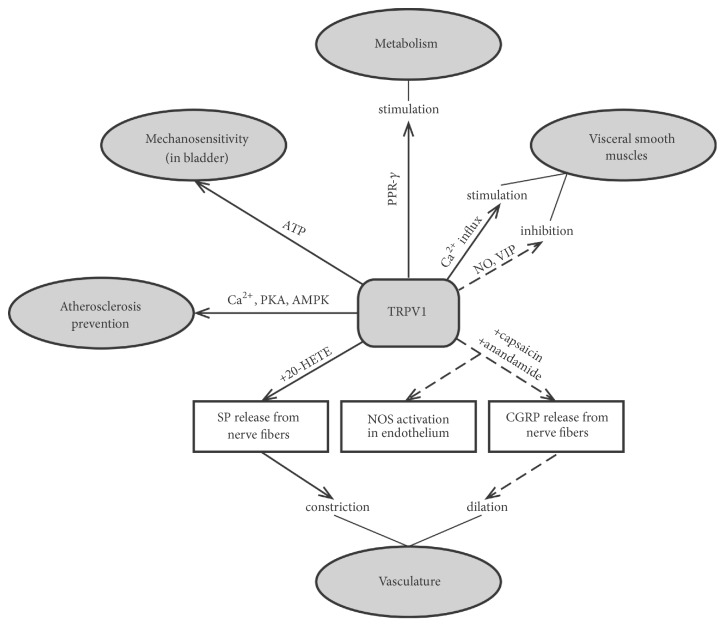
General outline of TRPV1 channels' role in signaling pathways that regulate vascular and visceral functions. TRPV1: transient receptor potential channel vanilloid family type 1; AMPK: AMP activated protein kinase; CGRP: calcitonin gene-related peptide, 20-HETE: 20-hydroxy-5, 8, 11, 14-eicosatetraenoic acid; NOS: NO synthase; PKA: protein kinase A; PPR-*γ*: peroxisome proliferator-activated receptor-*γ*; SP: substance P; and VIP: vasoactive intestinal polypeptide.

Different physiological functions and processes, described above, illustrate the variety of TRPV1 implications into the regulation of body functions and disease development. These are summarized in Figure 1.

## 5. Structural Relatedness of TRPV1 in Different Species and Animal Models of Human Disorders

In common with other TRP channels, TRPV1 channels when activated perform two main cellular roles; namely, most TRPs provide an additional entry route for Ca^2+^, while activation of these cation-selective channels invariable causes membrane depolarization, which allows cells expressing voltage-gated Ca^2+^ channels to trigger this additional powerful Ca^2+^ entry mechanism. However, notwithstanding such commonness, it is also important to consider some possible species-dependent structure-function differences, which may concern more subtle questions of channel regulation and which are worth considering in choosing the most appropriate animal model of human disease.

We have recently described some important species-related differences in gating properties of receptor-operated TRPC4 channel [123]. Regarding TRPV1, some important species structural differences also exist that may confer differences in biophysical and/or pharmacological properties of the channel. One striking example is chicken ortholog of TRPV1, which can be activated by heat and protons, but not by capsaicin [124]. To further address this issue, we have performed analysis of structural relatedness of TRPV1 in several species by focusing on UniProt data, for which experimental evidence at protein level exist. Multiple sequence alignment with CLUSTALW revealed the highest degree of sequence identity between mouse and rat TRPV1 (score 94.9881), while the lowest score was found for human and rat TRPV1 (84.9642). As mouse models of human disorders are widely used, it should be noted that human vs. mouse TRPV1 score is 86.174.

**Figure 2:**
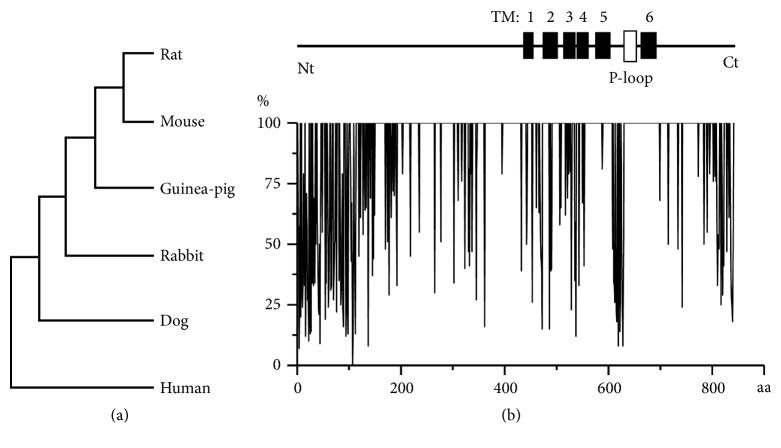
Species-related structural differences in TRPV1. (a) Phylogenetic tree constructed by CLUSTALW Multiple Sequence Alignments for several TRPV1 proteins (with accession numbers of protein sequences), namely, human (Q8NER1), rat (O35433), mouse (Q704Y3), dog (Q697L1), guinea-pig (Q6R5A3), and rabbit (Q6RX08) isoforms of TRPV1. (b) CLUSTALX 2.1 column scores for aa sequences in 6 mammalian TRPV1s shown in panel (a). A simplified protein topology is schematically shown at the top. TM: transmembrane domains. P-loop: pore-forming region.

TRPV1 structural relatedness in the 6 species is illustrated by the phylogenetic tree in Figure 2(a). Furthermore, Figure 2(b) shows CLUSTALX 2.1 column scores for amino acid (aa) sequences in these species. Notably, the most highly evolutionary conserved topological domains of TRPV1 include its transmembrane segments (TM1-6), and in particular TM5 (99.3%) and TM6 (100%), as well as pore-forming P-loop (100%), while most changes are found in intracellular N- (Nt) and C-termini (Ct) of the protein. These regions contain amino acid residues and sites important for regulating TRPV1 sensitivity via phosphorylation/dephosphorylation reactions and plasma membrane insertion, as well as binding sites for PI(4,5)P_2_ and calmodulin, which regulate channel activity. Six ankyrin repeats are contained within Nt, and at least some of these are involved in channel tetrameric assembly (reviewed by Bevan et al., [71]).

Thus, based on this analysis, we can propose that important species-dependent differences may exist regarding trafficking, membrane insertion, biophysical and pharmacological properties, and regulation (and especially sensitization by protein phosphorylation/dephosphorylation) of TRPV1. These should be considered in the context of the most appropriate animal model of a human disorder, warranting more research on these aspects of TRPV1 structure-function relations.

## 6. Concluding Remarks and Future Perspectives

While TRPV1 continues to attract the main interest of both academic researchers and pharmaceutical industry as “the pain receptor,” accumulating evidence suggests that it is a widely expressed channel protein that subserves an amazingly wide array of very different functions not only in the nervous system, but also in most, if not all, peripheral tissues. It is thus not surprising that TRPV1 altered expression and/or function has been found in multiple disorders, such as epilepsy, depression, schizophrenia, Alzheimer's disease, pulmonary hypertension, atherosclerosis development, asthma and chronic cough, irritable bowel syndrome, overactive bladder, diabetes, and obesity, as reviewed here.

In theory, pharmacological modulators of TRPV1 activity may thus present many novel and exciting opportunities for the treatment of these disorders. However, there is increasingly cautious optimism about such therapeutic interventions. Indeed, many challenging questions remain to be answered, such as

- Is altered TRPV1 expression and/or function the main culprit in a certain human disorder?
- Are animal models correctly represent all the main features of human disease considering the above discussed species-related structural, and likely functional, differences?
- Since the same pathological condition can alter TRPV1 expression, how such vicious cycle can be interrupted?
- Since TRPV1 and its various splice variants can form heterotetrameric complexes, what are functional and pharmacological consequences of such interactions?

Finally, and perhaps most importantly, new strategies of treatment will have to address the key problem of specific targeting of this multifunctional channel protein in the areas with pathological condition with no or minimal effect on its function in healthy tissues.
